# Optimizing internal biosecurity on pig farms by assessing movements of farm staff

**DOI:** 10.1186/s40813-023-00310-4

**Published:** 2023-04-15

**Authors:** Elise Bernaerdt, Inmaculada Díaz, Carlos Piñeiro, Miquel Collell, Jeroen Dewulf, Dominiek Maes

**Affiliations:** 1 Unit of Porcine Health Management, Department of Internal Medicine, Reproduction, and Population Medicine, Faculty of Veterinary Medicine, Ghent University, Salisburylaan 133, 9820 Merelbeke, Belgium; 2 PigCHAMP Pro Europa, C. Dámaso Alonso 14, 40006 Segovia, Spain; 3 MSD Animal Health, C. de Josefa Valcárel 38, 28027 Madrid, Spain; 4 Veterinary Epidemiology Unit, Department of Internal Medicine, Reproduction, and Population Medicine, Faculty of Veterinary Medicine, Ghent University, Salisburylaan 133, 9820 Merelbeke, Belgium

**Keywords:** Pig production, Internal biosecurity, Farm staff movements, Working lines

## Abstract

For internal biosecurity, it is important to separate different age groups in a pig farm and to stick to specific working lines when visiting the barns. Currently, there is no research on the movements of farm staff on pig farms. The objectives of this observational study were to assess movements of farm staff on pig farms, to assess risky movements and to investigate whether movements differ according to time (week of the batch farrowing system (BFS) and weekday vs. weekend) and unit (farrowing, gestation/insemination, nursery, and fattening unit). Five commercial sow farms participated and on each farm, an internal movement monitoring system was installed. Detection points were installed throughout the farm and workers had to wear a personal beacon. Movement data were collected from 1 December 2019 until 30 November 2020. The following sequence of movements was considered as safe: (1) dressing room, (2) farrowing, (3) gestation/insemination, (4) nursery, (5) fattening, (6) quarantine, and (7) cadaver storage. Movements in the opposite direction were considered as risk, unless a dressing room was visited in between. The total number of movements differed according to week of the BFS, and was highest in insemination and farrowing week. The percentage of risky movements was influenced by week of the BFS for two farms, and was highest around weaning. The percentage of risky movements varied between farms and ranged from 9 to 38%. There were more movements on a weekday compared to a weekend day. There were more movements towards the farrowing and gestation/insemination unit in insemination and farrowing week compared to other weeks of the BFS, but week of the BFS had no impact on movements towards nursery and fattening unit. This study showed that there were a lot of (risky) movements on pig farms and that these movements varied according to week of the BFS, day of the week, and unit. This study creates awareness, which could be a first step in optimizing working lines. Future research should focus on why certain risky movements occur and how these can be avoided to achieve better biosecurity and higher health status on farms.

## Introduction

Infections with specific pathogens commonly occur in pig farms and may result in major economic losses for the farmer. Such pathogens are transmitted through different routes, either directly via contact with infected animals or indirectly via people, semen, manure, rodents, aerosol, feed, water, or fomites [1]. Biosecurity measures on a farm aim to limit or even prevent the transmission of pathogens. All measures aiming to reduce the risk of pathogen introduction on a farm are grouped as external biosecurity measures, while those aiming to reduce the spread of pathogens within a farm are grouped as internal biosecurity measures. The implementation of biosecurity measures has multiple benefits, such as a reduced disease incidence and less antimicrobial usage [2], better production parameters [3, 4], and improved farm profitability [2, 5]. A previous study in France has shown that farm structure and working lines were significantly associated with a lower antimicrobial usage [6].

The European Animal Health Law emphasizes the importance of biosecurity to prevent the spread of infectious diseases to and within farms. Farm staff should acquire the appropriate knowledge and they should take action to minimize the spread of pathogens by working according to the correct working lines [7]. Each visit to a pig farm from both farm staff and visitors should start in a dressing room, where farm-specific clothing and footwear can be put on and where hands can be properly washed [8, 9]. Additional dressing rooms for each animal category could further reduce the risk of pathogen transmission [9]. Furthermore, farm staff should follow a specific sequence in visiting the units with different animal categories. Younger animals are more susceptible to various pathogens due to decreased maternal immunity while they have not yet developed a mature active immunity, whereas older animals are considered to be more robust but at the same time they may also harbor more infectious agents due to previous infections. Often these will remain unnoticed as a result of subclinical infection status. Therefore, movements or daily work should ideally be performed from young to old and from healthy to sick animals, thus according to the following sequence: (1) dressing room, (2) farrowing unit, (3) gestation/insemination unit, (4) nursery unit, (5) fattening unit, (6) quarantine unit, and (7) cadaver storage [9, 10]. Movements in the opposite direction are considered risky as they may cause pathogen transmission. Therefore, biosecurity measures aim at separating different age groups as much as possible. If these (virtual) separations are breached in specific units or on specific time points, then the overall biosecurity goes down and the efforts made in the other units or on different time points may be nullified.

A way to increase the awareness and motivation of pig farmers is to evaluate the biosecurity in pig farms [11]. The most common way to evaluate biosecurity is an assessment based on scores, such as Biocheck.UGent™ [12]. Although these scoring systems are good for creating awareness, they do not evaluate every component of the biosecurity in detail as this would make the overall assessment too complex and laborious. In the Biocheck.UGent™ questionnaire for pigs, there are two questions related to movements of farm staff, namely: (1) Are diseased pigs consistently handled/visited after the healthy ones? and (2) Is all the farm work performed from younger pigs to older pigs [12]? To verify if these conditions were applied consistently, Precision Livestock Farming (PLF) could be used. PLF is a concept in animal production where modern technologies, such as sensors and algorithms, are used to automatically gather data about the animals in order to optimize management practices [13, 14]. Some examples in pig production are electronic feeders to register feed intake by the animals [15], sensors that register estrus behavior in sows to optimize the moment of insemination [16], real-time sound analysis for health monitoring [17], and sensors monitoring the stable climate 24/7 [18]. Another example is the real-time internal movement monitoring system Biorisk® developed by PigCHAMP Pro Europa. This sensor is not for the animals, but for farm staff. The system can be used to monitor the working lines of farm staff in pig farms and to preserve good biosecurity routines [19].

Currently, there is no information on the movements of farm staff in pig farms. The general objective of the present study was to investigate the movements of staff in commercial pig farms. Possible movement differences according to time (week of a batch farrowing system (BFS) and day of the week) and unit (farrowing, gestation/insemination, nursery and fattening unit) in the farm were also investigated.

## Materials and methods

### Study design

This observational study was performed from 1 December 2019 until 30 November 2020. Farms were selected based on willingness to participate and to install wireless internet connection in all barns. Five commercial sow farms participated in the study and their characteristics are described in Table 1. In a BFS, the main tasks on a farm, i.e., weaning, insemination, and farrowing, are divided over different weeks. For farm A, working in a 3-week system, the following terminology for the different weeks will be used: weaning (the week in which a group of sows is weaned), insemination (the week in which a group of sows is inseminated), and farrowing (the week in which a group of sows farrow). For farms B, C, D, and E, working in a 4-week system, the following terminology will be used: weaning (the week in which a group of sows is weaned), insemination + farrowing (the week in which a group of sows is inseminated, and another group of sows is planned to farrow), “no main activities 1” (the week after farrowing week, when suckling piglets are handled in the farrowing unit, e.g., iron injection), and “no main activities 2” (the week where the nursery pigs are moved to the fattening unit).

**Table 1:** Characteristics of the five farms that participated in the study

	Farm A	Farm B	Farm C	Farm D	Farm E
Type of farm	Farrow-to-finish	Farrow-to-finish	Farrow-to-finish	Farrow-to-wean	Farrow-to-finish
Batch farrowing system (…week system)	3	4	4	4	4
Number of full-time employees	1	1	2	5	3
Sow breed	PIC	TN70	Hypor	Danbred	Danbred
Number of animals					
Sows	280	480	300	780	600
Nursery pigs	600	2000	1450	2500	3600
Fatteners	2500	1200	480	0	1000
Live born piglets per litter	13.1	14.6	14.1	17.5	16.6
Pre-weaning mortality (%)	12	13.4	13.7	17	7.9
Number of rooms					
(number of detection points)					
Dressing room	1 (1)	1 (1)	2 (2)	2 (2)	1 (1)
Farrowing unit	8 (3)	10 (10)	3 (3)	7 (3)	1 (2)
Gestation/insemination unit	2 (2)	7 (6)	3 (4)	8 (8)	6 (7)
Nursery unit	8 (3)	6 (1)	20 (4)	12 (3)	7 (5)
Fattening unit	11 (11)	6 (3)	6 (1)	0 (0)	5 (3)
Quarantine unit	2 (1)	1 (1)	1 (1)	2 (2)	4 (1)
Cadaver storage	1 (1)	1 (1)	1 (0)	1 (1)	1 (1)
Shower in the dressing room	No	No	No	Yes	Yes
Separate clothing and footwear for different units	No	No	No	Yes	Yes
Measures needed to enter the quarantine unit	Boots in disinfection bath	None	Changing boots	Changing coverall and boots	Changing coverall and boots
Location of cadaver storage	Close to the barns	Near to public road (far from the barns)	Near to public road (far from the barns)	Near to public road (far from the barns)	Near to public road (far from the barns)
Biosecurity scores (%)					
Total	60	67	81	72	86
External	66	76	87	77	83
Internal	54	57	74	66	88

For the housing of the animals, the terms room, unit, and barn will be used. Room refers to a room where animals of the same category are present, except for the farrowing room in which both sows and suckling piglets are present. A unit can consist of one or more rooms; and a barn can consist of one or more units. In some cases, a barn consists of different types of units, e.g., a barn with a farrowing and a gestation/insemination unit.

**Fig. 1:**
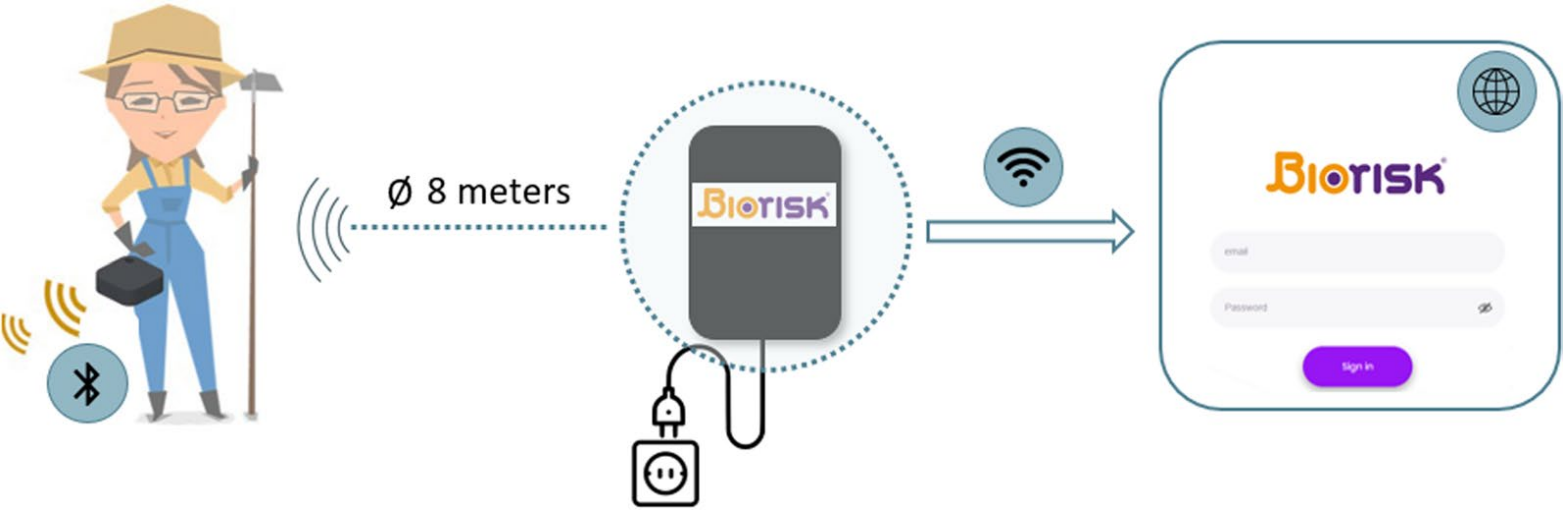
Graphical representation of the Biorisk® system developed by PigCHAMP Pro Europa

In all five farms, the internal movement monitoring system Biorisk® developed by PigCHAMP Pro Europa was installed [19]. For this purpose, detection points were installed in the rooms of the different animal categories on the farm. The detection points had a range of eight meters, and were installed in such a way to cover all rooms and entrances of the farm. In some units, only one detection point was needed, while in others more than one was needed to ensure all rooms were covered. A time filter was set for each detection point to avoid wrong detections in places where a detection point was too close to a corridor. All farm workers had to wear a small personal Bluetooth® transmitter, called a beacon. This transmitter sent a signal to the detection points, allowing the detection of the movements of farm staff. A wireless internet connection was needed to send data from the detection points to an online platform for further analysis (Fig. 1). The number of detection points and farm workers contributed to the total number of movements on a farm.

The biosecurity status of the farms was determined using the risk-based biosecurity quantification tool Biocheck.UGent™. This tool enables an objective quantification of the biosecurity status of the farm. Based on a questionnaire a score between 0 and 100% is given in different categories. Zero means a lack of any biosecurity measures, while 100 means perfect biosecurity [12]. The overall biosecurity scores and the subtotal for the external and internal biosecurity are shown in Table 1.

**Fig. 2:**
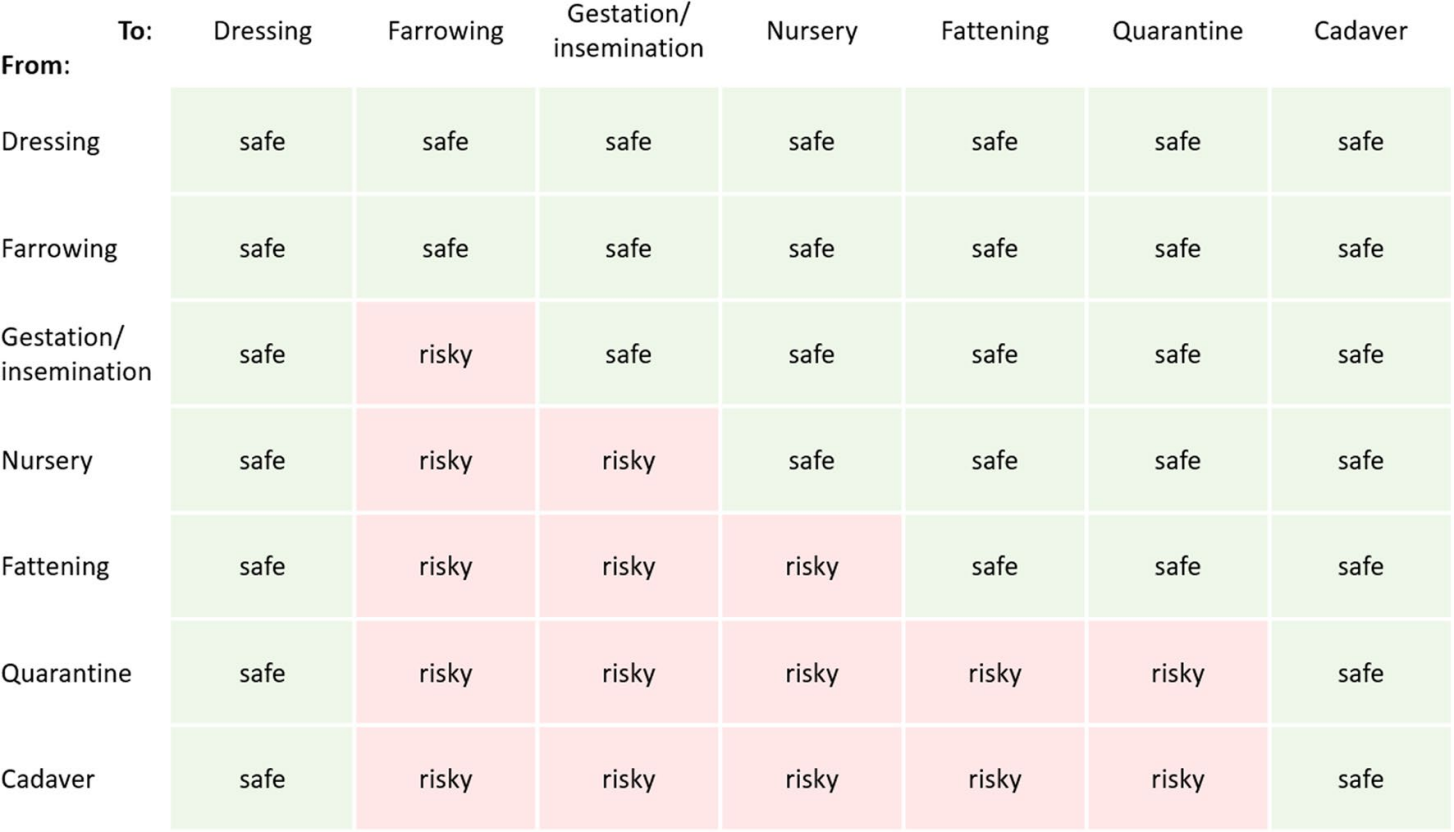
Definition of safe and risky movements by persons in the five farms included in the study

Movements from the farrowing unit to the gestation/insemination unit, followed by the nursery unit, fattening unit and finally, the quarantine unit and cadaver storage, were considered as safe movements [9]. Movements in the opposite direction were considered as risk, unless a dressing room was visited in between (Fig. 2). Movements between rooms of the same type, e.g., farrowing to farrowing room, were also considered as safe movements, except for a movement from a quarantine to another quarantine room. In total, 49 different movements could be distinguished, 33 of them were considered as safe and 16 as risky. Farm C did not have a power socket available at the cadaver storage; therefore, in this farm, movements from and to the cadaver storage could not be taken into consideration.

### Data analysis

All statistical analyses were performed using IBM® SPSS® Statistics for Windows Version 28 (IBM Corp., Armonk, N.Y., USA). Descriptive statistics were performed for the continuous variables. Normality distribution was analyzed graphically via histograms and Q–Q plots. All movement data were not normally distributed; therefore, the median, minimum, and maximum values were used.

Since the number of detection points and the number of animals varied between the farms, we standardized the total number of daily movements to allow comparison between the farms. For movements towards the farrowing and the gestation/insemination unit, the movements were standardized per detection point and per 100 sows. For movements towards the nursery and fattening unit, the movements were standardized per detection point and per 1000 nursery or fattening pigs.

A non-parametric independent samples Kruskal–Wallis test with Bonferroni correction was used to analyze potential differences in the total number of daily movements, percentage of risky movements, movements towards specific units within the farm, and number of movements towards specific units standardized by farm size between the different weeks of the BFS. For the latter, only the farms working in a 4-week BFS (B–E) were included in the analysis. Farm D was not included in the analysis of the movements towards the fattening unit, since there were no fattening pigs present on this farm. A non-parametric independent-samples Mann–Whitney U test was used to analyze potential differences in the total number of movements and percentage of risky movements between a weekday (Monday to Friday) versus a day on the weekend (Saturday and Sunday). *p-*values below 0.05 were considered to be statistically significant.

## Results

### Movements of farm staff: Biocheck.UGent and farm size

The Biocheck.UGent™ questionnaire for pigs was carried out in all farms. Specific attention was paid to the two questions related to movements of farm staff. Farms C and E stated that diseased pigs were consistently handled/visited after the healthy ones. Farms A, C, D, and E stated that all the farm work was performed from younger to older pigs.

The overall median percentages of risky movements on the farms were 11%, 33%, 36%, 15%, and 14%, and the numbers of sows were 280, 480, 300, 780, and 600 for farms A, B, C, D, and E, respectively. The lowest percentage of risky movements was seen on the smallest farm (farm A). There were a few more risky movements on the largest farms (farms D and E), and the highest percentage of risky movements was seen on the medium-sized farms (farms B and C).

### Movements of farm staff according to the week of the batch farrowing system

Table 2 shows the total number of daily movements and the percentage of risky movements in the different weeks of the BFS. The total number of movements significantly differed according to the week of the BFS for farms B (*p* = 0.005), C (*p* < 0.001), D (*p* < 0.001), and E (*p* = 0.029), with the highest number of total movements during insemination and farrowing week, followed by weaning week. The percentage of risky movements significantly differed according to the week of the BFS for farms C (*p* = 0.014) and D (*p* = 0.020), with the highest percentages in the weaning week.

**Table 2:** The median (min.–max.) number of daily movements and the percentage of risky movements in the different weeks of the batch farrowing system in farm A, working in a 3-week system, and farms B, C, D, and E, working in a 4-week system

	Total number of movements	Percentage of risky movements
*Farm A*		
Weaning	32^a^ (2–76)	11^a^ (0–30)
Insemination	32^a^ (9–75)	9^a^ (0–32)
Farrowing	33^a^ (13–90)	11^a^ (0–35)
*Farm B*		
Weaning	32^ab^ (2–633)	33^a^ (0–52)
Insemination + farrowing	53^a^ (2–348)	33^a^ (0–46)
No main activities 1	33^b^ (1–349)	33^a^ (0–45)
No main activities 2	38^b^ (2–320)	32^a^ (0–50)
*Farm C*		
Weaning	58^a^ (21–244)	38^a^ (10–46)
Insemination + farrowing	64^a^ (14–236)	35^b^ (17–47)
No main activities 1	49^b^ (14–166)	36^ab^ (20–44)
No main activities 2	41^c^ (13–103)	36^ab^ (8–50)
*Farm D*		
Weaning	71^ab^ (10–247)	16^a^ (6–27)
Insemination + farrowing	85^a^ (8–210)	14^ab^ (4–27)
No main activities 1	66^bc^ (2–173)	15^ab^ (0–32)
No main activities 2	52^c^ (7–273)	13^b^ (0–28)
*Farm E*		
Weaning	43^ab^ (5–487)	14^a^ (0–44)
Insemination + farrowing	57^a^ (2–351)	10^a^ (0–60)
No main activities 1	31^ab^ (1–629)	12^a^ (0–40)
No main activities 2	35^b^ (1–544)	14^a^ (0–40)

^abc^Within each farm and within a column, values with different superscript differed significantly (*p* < 0.05)

### Movements of farm staff during weekdays versus days during the weekend

Possible differences in movements between a weekday versus a day on the weekend were investigated (Table 3). On all farms, there was a higher total number of movements on a weekday than on a weekend day. This difference was statistically significant for all farms, except for farm C. On farms B, C, and D, there was a significant difference in the percentage of risky movements on a weekday than on a weekend day. On farms B and D there were less risky movements during the weekend, while on farm C there were more risky movements during the weekend.

**Table 3:** The median (min.–max.) number of daily movements and the percentage of risky movements on a weekday and a weekend day for the different farms (*n* = 5)

Farm	Total number of daily movements			Percentage of risky movements		
	Week	Weekend	*p*-value	Week	Weekend	*p*-value
A	37 (2–90)	24 (10–51)	< 0.001*	10 (0–32)	11 (0–35)	0.216
B	45 (2–633)	27 (1–349)	< 0.001*	33 (0–50)	31 (0–52)	0.046^*^
C	52 (14–236)	51 (13–244)	0.242	36 (10–50)	37 (8–44)	0.041^*^
D	80 (10–273)	34 (2–125)	< 0.001*	15 (0–29)	13 (0–32)	0.003^*^
E	50 (1–629)	31 (1–544)	0.001*	13 (0–60)	11 (0–60)	0.661

*The *p*-value is considered statistically significant (*p* < 0.05)

### Movements of farm staff towards the farrowing and gestation/insemination unit

Table 4 shows the total number of movements and the percentage of risky movements towards the farrowing and gestation/insemination unit. The total number of movements and the percentage of risky movements towards the farrowing unit significantly differed for the different weeks of the BFS for all the farms (*p* < 0.05), except for the risky movements on farm A (*p* = 0.403) and farm E (*p* = 0.259). There were more movements on a day in the insemination and farrowing week. The percentage of risky movements was highest during the weaning week.

**Table 4:** Median (min.–max.) number of daily movements (total movements and percentage of risky movements) towards the farrowing unit and the gestation/insemination unit in the different weeks of the batch farrowing system in farm A, working in a 3-week system, and farms B, C, D, and E, working in a 4-week system

	Total number of movements (*n*)		Percentage of risky movements (%)	
	Farrowing	Gestation/insemination	Farrowing	Gestation/insemination
*Farm A*				
Weaning week	4^a^ (1–12)	4^a^ (1–17)	40^a^ (0–100)	33^b^ (0–100)
Insemination week	2^b^ (1–8)	5^a^ (1–16)	33^a^ (0–100)	16^a^ (0–100)
Farrowing week	4^a^ (1–11)	4^a^ (1–11)	33^a^ (0–100)	20^a^ (0–100)
*Farm B*				
Weaning	6^b^ (1–158)	16^ab^ (1–344)	100^a^ (0–100)	20^b^ (0–100)
Insemination + farrowing	15^a^ (1–169)	25^a^ (2–105)	94^b^ (0–100)	13^a^ (0–45)
No main activities 1	7^b^ (1–94)	14^b^ (1–169)	94^b^ (0–100)	21^b^ (0–100)
No main activities 2	8^b^ (1–36)	16^b^ (1–151)	100^b^ (0–100)	21^b^ (0–100)
*Farm C*				
Weaning	22^a^ (6–95)	19^a^ (3–103)	89^a^ (33–100)	6^ab^ (0–57)
Insemination + farrowing	23^a^ (3–94)	23^a^ (1–123)	88^a^ (50–100)	8^a^ (0–100)
No main activities 1	18^ab^ (4–70)	14^b^ (4–57)	83^b^ (50–100)	4^b^ (0–50)
No main activities 2	16^b^ (4–43)	14^b^ (2–37)	87^ab^ (25–100)	0^b^ (0–38)
*Farm D*				
Weaning	22^a^ (1–120)	28^a^ (4–97)	40^a^ (13–100)	8^ab^ (0–24)
Insemination + farrowing	39^b^ (3–86)	24^ab^ (1–77)	33^b^ (8–60)	6^a^ (0–29)
No main activities 1	27^a^ (2–87)	19^bc^ (1–78)	35^b^ (3–67)	7^a^ (0–30)
No main activities 2	13^c^ (2–34)	16^c^ (2–39)	36^b^ (0–63)	11^b^ (0–50)
*Farm E*				
Weaning	24^a^ (1–106)	8^a^ (1–49)	67^a^ (3–100)	33^a^ (0–100)
Insemination + farrowing	44^b^ (2–129)	7^ab^ (1–46)	45^a^ (0–100)	9^ab^ (0–100)
No main activities 1	24^ab^ (1–120)	5^b^ (1–48)	55^a^ (0–100)	5^b^ (0–100)
No main activities 2	19^a^ (1–147)	6^ab^ (1–46)	55^a^ (0–100)	0^b^ (0–100)

^abc^Within each farm and within a column, values with different superscript differed significantly (*p* < 0.05)

The total number of movements and the percentage of risky movements towards the gestation/insemination unit significantly differed for the different weeks of the BFS for all the farms (*p* < 0.05), except for farm A. Farm workers had more movements towards the gestation/insemination unit in insemination and farrowing week. For the percentage of risky movements towards the gestation/insemination unit, there was not one specific week of the 4-week system with more risky movements and there was some variation between the farms (Table 4).

### Movements of farm staff towards the nursery and fattening unit

Table 5 shows the total number of movements and the percentage of risky movements towards the nursery and fattening unit. Since farm D was farrow-to-wean, there were no movements towards the fattening unit. Regarding the movements towards the nursery unit, we found significant differences in the total number of movements in farm C (*p* < 0.001) and the percentage of risky movements in farm E (*p* < 0.001). The movements towards the fattening unit did not significantly differ between the weeks of the BFS.

**Table 5:** Median (min.–max.) number of daily movements (total movements and percentage of risky movements) towards the nursery unit and fattening unit in the different weeks of the batch farrowing system in farm A, working in a 3-week system, and farms B, C, D, and E, working in a 4-week system

	Total number of movements (*n*)		Percentage of risky movements (%)	
	Nursery	Fattening	Nursery	Fattening
*Farm A*				
Weaning week	2^a^ (1–9)	8^a^ (1–29)	0^a^ (0–100)	5^a^ (0–50)
Insemination week	2^a^ (1–7)	7^a^ (2–26)	0^a^ (0–100)	13^a^ (0–50)
Farrowing week	2^a^ (1–9)	7^a^ (2–33)	0^a^ (0–100)	7^a^ (0–33)
*Farm B*				
Weaning	2^a^ (1–11)	3^a^ (1–19)	0^a^ (0–100)	0^a^ (0–50)
Insemination + farrowing	1^a^ (1–8)	3^a^ (1–16)	0^a^ (0–100)	0^a^ (0–100)
No main activities 1	2^a^ (1–13)	3^a^ (1–13)	0^a^ (0–100)	0^a^ (0–50)
No main activities 2	1^a^ (1–19)	3^a^ (1–38)	0^a^ (0–100)	0^a^ (0–67)
*Farm C*				
Weaning	6^ab^ (1–30)	2^a^ (1–24)	0^a^ (0–77)	0^a^ (0–50)
Insemination + farrowing	6^b^ (1–19)	2^a^ (1–6)	0^a^ (0–40)	0^a^ (0–50)
No main activities 1	5^ac^ (1–16)	2^a^ (1–7)	0^a^ (0–33)	0^a^ (0–0)
No main activities 2	4^c^ (1–13)	2^a^ (1–6)	0^a^ (0–83)	0^a^ (0–100)
*Farm D*				
Weaning	7^a^ (1–47)	–	0^a^ (0–10)	–
Insemination + farrowing	6^a^ (1–33)	–	0^a^ (0–13)	–
No main activities 1	6^a^ (1–31)	–	0^a^ (0–0)	–
No main activities 2	8^a^ (1–134)	–	0^a^ (0–0)	–
*Farm E*				
Weaning	12^a^ (1–146)	3^a^ (1–23)	0^a^ (0–100)	0^a^ (0–100)
Insemination + farrowing	15^a^ (1–74)	3^a^ (1–9)	0^a^ (0–100)	0^a^ (0–100)
No main activities 1	9^a^ (1–269)	3^a^ (1–19)	0^a^ (0–100)	0^a^ (0–56)
No main activities 2	9^a^ (1–119)	3^a^ (1–17)	9^b^ (0–100)	0^a^ (0–100)

^abc^Within each farm and within a column, values with different superscript differed significantly (*p* < 0.05)

### Number of movements towards the different units standardized by farm size

In order to enable proper comparison between farms, the number of movements towards the different units were standardized by number of detection points and farm size. There was a significant effect of the week of the BFS on movements towards the farrowing unit (*p* < 0.001) and gestation/insemination unit (*p* < 0.001) (Table 6). Overall, most movements towards the farrowing unit were made in insemination and farrowing week and the least movements in “no main activities 2”, i.e., the week where nursery pigs are moved to the fattening unit. There were more movements towards the gestation/insemination unit in weaning or insemination and farrowing week compared to the weeks with no main activities. There was no significant effect of the week of the BFS on movements towards the nursery or fattening unit.

**Table 6:** Median (min.–max.) number of daily movements towards the different units standardized per detection point and per 100 sows for movements towards the farrowing and gestation/insemination unit and per 1000 nursery/fattening pigs for movements towards the nursery and fattening unit in the different weeks of the batch farrowing system in farms B, C, D, and E, working in a 4-week system

	Movements per detection point per 100 sows (*n*)		Movements per detection point per 1000 nursery/fattening pigs (*n*)	
	Farrowing	Gestation/insemination	Nursery	Fattening
*Farms B, C, D, E*				
Weaning	1.1^a^ (0.0–10.6)	0.6^a^ (0.0–11.9)	0.9^a^ (0.1–8.1)	1.1^a^ (0.3–12.5)
Insemination + farrowing	1.6^b^ (0.0–10.8)	0.6^a^ (0.0–10.3)	0.9^a^ (0.1–4.4)	1.1^a^ (0.3–12.5)
No main activities 1	1.1^ac^ (0.0–10.0)	0.4^b^ (0.0–5.9)	0.8^a^ (0.1–14.9)	1.1^a^ (0.3–14.6)
No main activities 2	0.8^c^ (0.0–12.3)	0.4^b^ (0.0–5.2)	0.7^a^ (0.1–17.9)	1.1^a^ (0.3–12.5)

^abc^Within a column, values with different superscript differed significantly (*p* < 0.05)

## Discussion

The present study elucidated differences in movements of farm staff according to week of the BFS and weekday versus weekend; and unit, namely towards farrowing, gestation/insemination, nursery and fattening unit. The following movements differed according to the week of the BFS: total number of daily movements (highest in insemination and farrowing week) and percentage of risky movements (highest in weaning week). There were more farm staff movements during a weekday, but the percentage of risky movements was for some farms higher and for others lower on a weekend day. The present study also gained more insight into movements towards the different units. There were more movements of farm staff towards the farrowing and gestation/insemination unit during insemination and farrowing week, compared to other weeks of the BFS. Movements towards the nursery and fattening unit did not differ according to the week of the BFS, except for the total number of movements towards the nursery in one farm and the percentage of risky movements towards the nursery unit in another farm.

According to the results of the Biocheck.UGent™ questionnaire, all farms except for farm B claimed to organize their work consistently starting with the young animals and then continuing the work in the older animals. However, the results of the present study did not confirm this, as a high percentage of risky movements was observed on the farms. This illustrates that monitoring the behavior of farmers is key to obtain accurate data, as farmers might not always provide the correct answer in observational studies. On larger farms, farmers should implement more biosecurity measures compared to smaller farms, because a larger number of animals also means that more animals can get sick and spread infections. Moreover, larger herds come more into contact with the outside world, e.g., by purchasing animals and livestock transport, increasing the risk of infection [20, 21]. In the present study, there were less risky movements on larger farms compared to medium-sized farms. On these large farms, there were more employees and it is possible that certain employees were only responsible for the work in certain units, resulting in less movements between the different units. Furthermore, previous studies in both pig [3, 22, 23] and cattle production [24, 25] have shown that biosecurity measures are better implemented in larger farms.

The total number of daily movements significantly differed according to the week of the BFS for farms B, C, D, and E, all working in a 4-week system. On farm A, working in a 3-week system, there was no difference in the total number of daily movements according to the week of the BFS. A possible explanation could be that the main activities on the farm, i.e., weaning, insemination, and farrowing, are more evenly spread in a 3-week system, leading to a more even distribution of the movements over the different weeks. It is also noteworthy that the percentage of risky movements was the lowest on the farm using the 3-week system. This may be explained by the fact that the 3-week system allows for a better organization of the work throughout the weeks. In a 4-week system, there is one week with two main activities which demand extra work, i.e., insemination and farrowing, which could have led to a peak in the number of daily movements in that specific week of the BFS. Also, in the weaning week there were many movements, likely because sows had to be moved from the farrowing to the gestation/insemination unit and piglets from the farrowing to the nursery unit.

On farms A, B, and E, there were no significant differences in the percentage of risky movements according to the week of the BFS, meaning that the farmers applied a consistent working routine irrespective of the specific week of the BFS. Although there were no significant differences, the percentage of risky movements was high in all farms. Median values ranged from 9 to 33%, indicating that farmers often do not adhere to the biosecurity standards. This implies that there is much room for improvement. On farms C and D, there were significantly more risky movements in the weaning week. This was expected, as around the time of weaning there may be a lot of risky movements from the nursery to the farrowing unit.

In general, there were more movements on a weekday versus a day on the weekend, and there are three possible explanations for this. The first one is that the BFS are well organized, and most activities are planned on weekdays. In both the 3- and 4-week system, weaning takes place on a Thursday, sows are inseminated on Monday, Tuesday, and Wednesday, and sows farrow on Thursday, Friday, and Saturday. Second, in farms D and E, there were several workers and they might have not been all present on the farm during the weekend, resulting in less movements on the weekend. Third, the work on the farm could be done more efficiently during the weekend to save time for other non-farm-related activities. The percentage of risky movements on a weekday was higher on farms B, D, and E, but on farm C this percentage was lower and more risky movements were made on a day during the weekend. On farm C, it is possible that the working routine was different during the weekend, and some extra violations to the correct working lines were made.

The total number of daily movements towards the farrowing and gestation/insemination unit was higher in the insemination and farrowing week, followed by the weaning week. The farrowing and gestation/insemination unit are places where much work is needed and farm staff possibly needed to visit these units more than once per day, for example for supervision at farrowing, treatment of suckling piglets, estrus detection and insemination. For the nursery and fattening unit, the number of movements was similar regardless of the week of the BFS. In the absence of specific problems, these units were probably just visited for feeding and routine check of the animals.

The percentage of risky movements towards the farrowing unit and in some farms towards the gestation/insemination unit was higher in the weaning week, but we found no significant differences in the percentage of risky movements towards the nursery and fattening unit. Furthermore, the percentage of risky movements towards the nursery and fattening unit was in general much lower compared to the percentage of risky movements towards the farrowing and the gestation/insemination unit. The nursery and fattening unit were visited less frequently and these visits were probably better organized during the working day, facilitating the implementation of biosecurity principles and as such reducing the risk of making a risky movement.

The number of movements standardized by farm size towards the farrowing and gestation/insemination unit significantly differed according to the week of the BFS, while this was not the case for the number of movements standardized by farm size towards the nursery and fattening unit. In Table 6, showing the number of movements standardized by farm size, values below one can be noticed. The values in the table were obtained because standardization was done to allow a comparison between the farms. In some units, multiple detection points were present in one room. Furthermore, the movements were standardized per 100 sows for movements towards the farrowing and gestation/insemination unit, and all farms had more than 100 sows, leading to these values below one.

To our knowledge, the internal movement monitoring system Biorisk® is the first technology to verify movements of farm staff. There are only a few studies available where this technology has been used. Geurts et al. (2018) studied the association between the number of risky movements and the prevalence of porcine reproductive and respiratory syndrome virus in a farm [26] and Black et al. [27] studied the association between movements and the number of weaned pigs per sow. In human medicine, similar technologies are already being used e.g., to monitor hand hygiene compliance in hospitals [28]. The internal movement monitoring system allows real-time detection of farm staff. All information is immediately processed on the online platform. However, there are also some limitations to the system. The detection points should be plugged into a socket at all times, and since the location of the detection points is crucial, in some farms extra sockets needed to be installed or extension cables were used. The range of the detection points is eight meters and goes through walls, so the time filter was needed to ensure that accidental detections were not registered. Furthermore, the system stands or falls by the dedication of the farm staff, as they should wear the beacon at all times. Regardless of these practical limitations, the internal movement monitoring system provided us with new and valuable information on the movements of farm staff in pig farms. The findings also complement the results of previous observational studies on biosecurity in pig farms.

Finally, besides the practical aspect, some ethical considerations are made. A previous study already raised some questions on data ownership, privacy, and cybersecurity concerning PLF [29]. The Biorisk® system aims to understand movements of farm staff in order to improve biosecurity, not to check individual farm workers or accusing them of outbreaks. In case of unauthorized use, the system could violate privacy of farm staff and might cause difficulties for larger farms to find external staff willing to work on the farm. Furthermore, these data should not be used by the government or quality assurance schemes to verify if animals were daily checked.

## Conclusions

The present study showed that there were a lot of (risky) movements on pig farms and that these movements varied according to week of the BFS, day of the week, and unit. This study creates awareness on movements of farm staff in pig farms, which is a first step in optimizing the working lines. It can lead to customized training for every farm based on objective data that show farm staff behavior and relating it to later health status and performance, aiming to promote a working culture of improving biosecurity, health and performance data-driven. Future research should provide insight into why specific risky movements occur and how these can be avoided to achieve a better biosecurity and higher health status on farms.

## Data Availability

The datasets used and/or analyzed during the current study are available from the corresponding author on reasonable request.
